# The predictive value of next generation sequencing for matching advanced hepatocellular carcinoma patients to targeted and immunotherapy

**DOI:** 10.3389/fimmu.2024.1358306

**Published:** 2024-04-11

**Authors:** Jiajia Du, Erlei Zhang, Zhiyong Huang

**Affiliations:** Hepatic Surgery Center, Tongji Hospital, Tongji Medical College, Huazhong University of Science and Technology, Wuhan, Hubei, China

**Keywords:** hepatocellular carcinoma, prognosis, NGS, immunotherapy, PD-L1

## Abstract

**Background:**

Targeted and Immunotherapy has emerged as a new first-line treatment for advanced hepatocellular carcinoma (aHCC). To identify the appropriate targeted and immunotherapy, we implemented next generation sequencing (NGS) to provide predictive and prognostic values for aHCC patients.

**Methods:**

Pretreatment samples from 127 HCC patients were examined for genomic changes using 680-gene NGS, and PD-L1 expression was detected by immunohistochemistry. Demographic and treatment data were included for analyses of links among treatment outcomes, drug responses, and genetic profiles. A prognostic index model for predicting benefit from treatment was constructed, taking into account of biomarkers, including *TP53*, *TERT*, PD-L1, and tumor mutation burden (TMB) as possible independent prognostic factors.

**Results:**

The multivariate Cox regression analyses showed that PD-L1≥1% (HR 25.07, 95%CI 1.56 - 403.29, p=0.023), TMB≥5Mb (HR 86.67, 95% CI 4.00 - 1876.48, p=0.004), TERT MU (HR 84.09, 95% CI 5.23 - 1352.70, p=0.002) and TP53 WT (HR 0.01, 95%CI 0.00 - 0.47, p=0.022) were independent risk factors for overall survival (OS), even after adjusting for various confounders. A prognostic nomogram for OS was developed, with an area under the ROC curve of 0.91, 0.85, and 0.98 at 1-, 2-, and 3- year, respectively, and a prognostic index cutoff of 1.2. According to the cutoff value, the patients were divided into the high-risk group (n=29) and low-risk group (n=98). The benefit of targeted and immunotherapy in the low-risk group was not distinguishable according to types of agents. However, treatment of Atezolizumab and Bevacizumab appeared to provide longer OS in the high-risk group (12 months vs 9.2, 9, or 5 months for other treatments, p<0.001).

**Conclusion:**

The prognostic model constructed by PD-L1, TMB, *TERT*, and *TP53* can identify aHCC patients who would benefit from targeted and immunotherapy, providing insights for the personalized treatment of HCC.

## 1 Introduction

Hepatocellular carcinoma (HCC) is the most common liver cancer and the fourth leading cause of cancer-related death worldwide (1). Most patients are diagnosed at an advanced stage with chronic liver disease and cannot receive surgical treatment (2). In recent years, targeted and immunotherapy has brought hope to patients with advanced hepatocellular carcinoma (aHCC). Compared with Sorafenib, The IMbrave150 trial showed that the combination of Atezolizumab and Bevacizumab prolonged overall survival (OS) (19.2 mo vs. 13.4 mo, HR=0.66, 95% CI=0.52-0.85, P=0.0009) and progression-free survival (PFS) (6.9 mo vs. 4.3 mo, HR=0.65, 95% CI=0.53-0.81, P=0.0001) (3). Atezolizumab and Bevacizumab are FDA-approved for patients with unresectable or metastatic HCC who have not received previous systemic therapy (3). Pembrolizumab (4), Nivolumab (5), and others have also been approved by the FDA for HCC patients previously treated with Sorafenib.

With the rapid development of metagenomic sequencing technology, HCC has been shown to be significantly heterogeneous. Not all HCC patients can benefit from targeted and immunotherapy, and its high treatment resistance and poor prognosis pose challenges to systemic treatment (6). The objective response rate (ORR) of Atezolizumab combined with Bevacizumab is only about 30% (7). In addition, approximately 5%-30% of patients develop grade ≥3 immune-related adverse events (irAEs). To optimize the use of targeted and immunotherapy in HCC, the development of predictive biomarkers has become increasingly important.

Next-generation sequencing (NGS) can analyze the comprehensive information of the cancer genome and transcriptome on a large scale, thereby rapidly identifying potential driver gene events in cancer, especially potential molecular therapeutic targets (8). In non-small cell lung cancer (NSCLC), genetic biomarkers have been shown to predict the efficacy of targeted therapies, especially those targeting specific tyrosine kinase receptors and immune checkpoint inhibitors (9). Genetic testing also plays a crucial role in guiding the correct treatment of breast cancer, especially the stratification of patients (10). However, biomarkers that are useful for targeted and immunotherapy prediction in other cancers, such as programmed death ligand 1 (PD-L1) and tumor mutation burden (TMB), have no established predictive role in HCC.

In a multi-cohort study, previously treated, advanced HCC patients treated with combination therapy of PD-L1 inhibitor Durvalumab and anti-VEGFR2 antibody Ramucirumab, the high PD-L1 subgroup showed longer OS and PFS, compared with low PD-L1 subgroup (11). In CheckMate 459 trial, advanced HCC patients with baseline PD-L1 expression ≥1% had a higher ORR in Nivolumab group (28% vs 12%), compared with PD-L1<1% (12). It has been shown that higher TMB is associated with better tumor responsiveness to PD-1/PD-L1 immunotherapy in a variety of tumors, including HCC (13). However, several studies have shown that level of the TMB in HCC is relatively low (mTMB =4.08 mutations/Mb) (14). *TP53* is the most frequently mutated tumor suppressor gene in HCC. *TP53* gene aberrations are associated with specific IFN-γ gene signatures, higher Foxp3+ Treg infiltration and lower CD8+ T cell infiltration in HCC (15). *TERT* mutation and serum AFP≥400 ng/mL were independent predictors of poor OS in advanced HCC patients treated with Atezolizumab combined with Bevacizumab (16).

A single gene has limited accuracy in predicting response to targeted and immunotherapy. The study aims to construct a multi-gene prediction model, demonstrate the clinical application of NGS in patients with advanced HCC, and explore the relationship between response to first-line targeted and immunotherapy and genomic biomarkers.

## 2 Materials and methods

### 2.1 Patients selection

This retrospective study enrolled a total of 151 patients diagnosed with unresectable advanced HCC who underwent targeted and immunotherapy at Tongji Hospital in Wuhan between November 2020 and October 2023. Baseline blood samples and clinical data were collected from these patients. The study adhered to the guidelines outlined in the Helsinki Declaration and received approval from the Tongji Hospital’s Ethics Committee (TJ-IRB20230866). All subjects signed an informed consent form before tissue or blood collection and were informed about the use of the samples and the test results.

**Figure 1:**
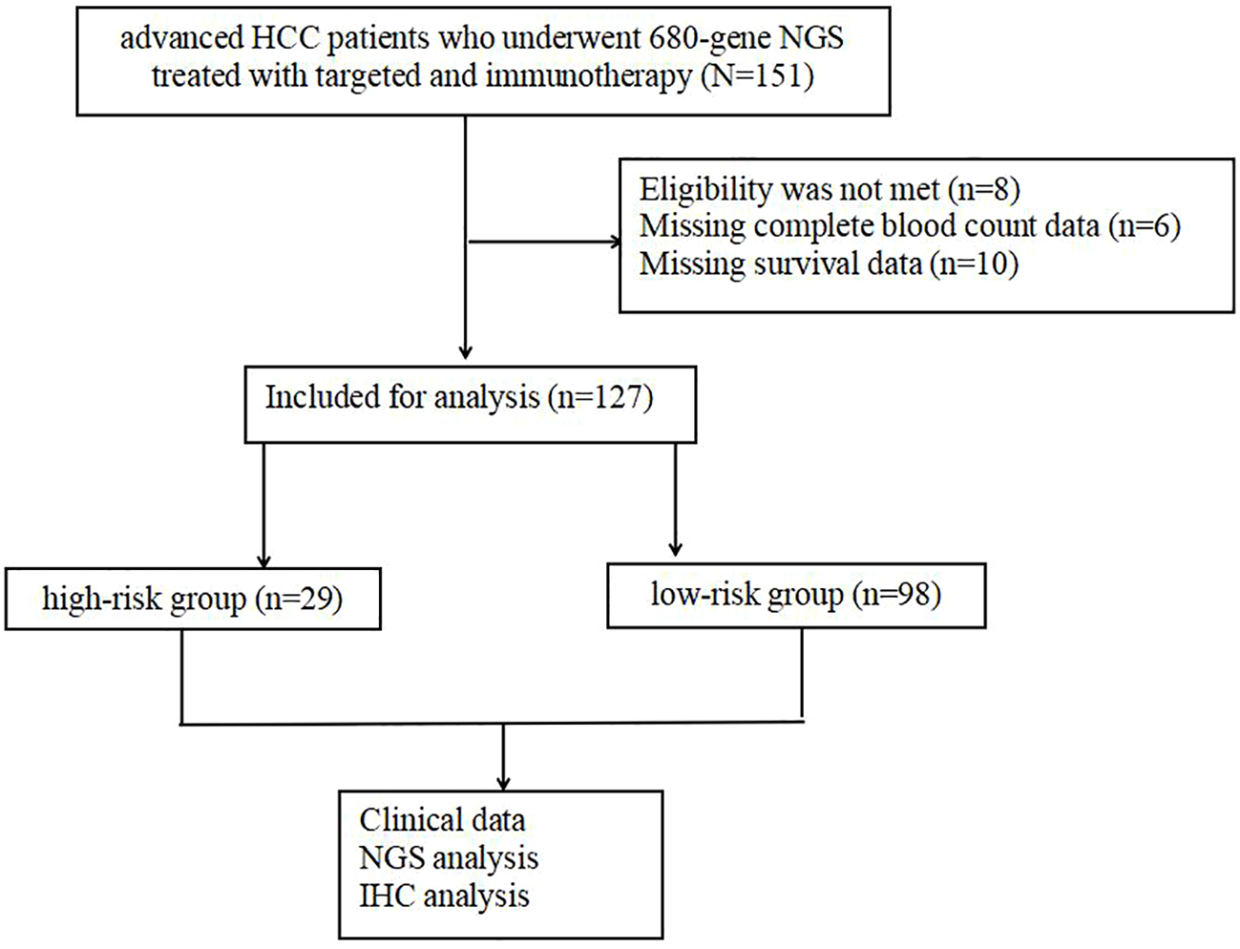
Data collected on the diagnosis of advanced HCC patients who underwent NGS and IHC.

Out of these, 24 individuals were excluded due to incomplete data, leaving a total of 127 individuals in the final analysis (**Figure 1**). The medical records collected included patient demographics, HCC etiology, Eastern Cooperative Oncology Group (ECOG) performance score, blood indicators (routine blood, liver function, and alpha-fetoprotein [AFP]), imaging data (maximum tumor diameter, number of metastases, vascular lymph node metastases, cirrhosis, and fatty liver), Child–Pugh class, Barcelona Clinic Liver Cancer (BCLC) stage, and pathological indicators (MVI and tumor differentiation).

The inclusion criteria were described below: 1) age 18–75 years; 2) expected survival >6 months, and ECOG score 0–1; 3) Child–Pugh 5–7 points; 4) BCLC stage: B and C; 5) neutrophil ≥1.5 × 10^9^/L, lymphocyte ≥0.5 × 10^9^/L, hemoglobin ≥90 g/L, and platelet ≥75 × 10^9^/L; 6) aspartate aminotransferase and alanine aminotransferase ≤3× the upper limit of normal value (ULN) and total bilirubin ≤3× the ULN; 7) international normalized ratio ≤1.5× the ULN; 8) creatinine ≤1.5× the ULN; 9) follow-up local treatments, such as radiation therapy (RT), transcatheter arterial chemoembolization (TACE), hepatic arterial infusion chemotherapy (HAIC) and radiofrequency ablation (RFA) are permissible.

The exclusion criteria were as follows: 1) previous local treatment and systematic treatments (chemotherapy, targeted therapy, immunotherapy); 2) individuals received antibiotics, hormones, immunosuppressants, aspirin, and atorvastatin 1 month before or during targeted therapy and immunotherapy; 3) acute inflammation, diabetes, hyperlipidemia, kidney disease, rheumatic disease, thyroid disease, autoimmune disease, or immune deficiency; 4) distant metastasis (except lymph node metastasis); and 5) malignancy with other sources.

### 2.2 Study design

All unresectable patients underwent liver tumor biopsy before treatment. All tumor samples were reviewed and confirmed to be HCC by at least two pathologists. After pathological examination, one part of the tumor tissue was frozen in a -80 refrigerator, and the other part was formalin-fixed and paraffin embedded to become tissue block sections (5-20μm).

### 2.3 680-gene NGS sequencing

#### 2.3.1 DNA extraction

Genomic DNA from fresh HCC tumors and patient- matched NATs (normal adjacent tissues) were extracted using the QIAamp DNA FFPE tissue kit (Qiagen). Extracted DNA was then quantified by Qubit 3.0 (Thermo Fisher Scientific, Inc., Waltham, MA, USA), in accordance with manufacturer’s instructions.

#### 2.3.2 Library construction

DNA was sheared using enzyme dsDNA Fragmentase (New England BioLabs, Inc., Ipswich, MA, USA). Size selection of the DNA fragments (150-250 bp) was then performed using Ampure XP beads (Beckman Coulter, Inc., Brea, CA, USA), which has the additional benefits of higher recovery and greater speed. DNA fragments were used for library construction using the KAPA Library Preparation kit (Kapa Biosystems, Inc., Wilmington, MA, USA) according to the manufacturer’s protocol. Agencourt AMPure XP beads (Beckman Coulter, Inc., Brea, CA, USA) were used for all the cleanup steps. End repair and 3’-end A-tailing were performed following DNA fragmentation. The purity and concentration of the DNA fragments were assessed using the Qubit 3.0 fluorometer and the Qubit dsDNA HS Assay kit.

#### 2.3.3 Targeted capture

Targeted capture was performed using a custom set of biotinylated DNA probes which contain 680 cancer-related genes encompassing 2.16 Mb (Roche NimbleGen). The hybridization of the amplified sample libraries and the SeqCap EZ Library was used according to the manufacturer’s protocol for 16-20h at 47°C. After hybrid selection, the captured DNA fragments were amplified with 12 to 14 cycles of PCR using 1× KAPA HiFi Hot Start Ready Mix and Post-LM-PCR Oligos in two separate 50 μL reactions. The reactions were then pooled and purified by Agencourt AMPure XP beads.

#### 2.3.4 Library denaturing, diluting and sequencing

Multiplexed libraries were denatured by Tris-HCl and diluted by 0.2N NaOH according to the manufacturer’s protocol (Illumina). Then the libraries were sequenced using 150-bp paired-end runs on an Illumina NovaSeq 6000 system (Illumina).

### 2.4 Immunohistochemistry

Paraffin sections were blocked with 10% goat serum albumin (AR1009, Wuhan Doctor De Bioengineering Co., LTD.) in PBS for 30 min and then incubated with PD-L1 antibody (ab213524, ABbcam, Wuhan, China). Goat anti-rabbit IgG-HRP (ab205718, ABbcam, Wuhan, China) was used as the secondary antibody, and the slides were observed under a microscope after DAB coloration.

### 2.5 Evaluation of PD-L1, TMB,*TERT* and *TP53*

Serial sections of stained tumor tissue were independently examined by two investigators, including a pathologist. To compare the intensity of cell staining for PD-L1, cells in serial sections were evaluated microscopically (x200 magnification). We selected three representative fields, each of which identified any expression of PD-L1 in 100 tumor cells. For PD-L1 IHC analysis, TPS cut-off value were set at 1% to distinguish between presence and absence of PD-L1 expression.

TMB is defined as the number of nonsynonymous somatic mutations contained within an average 1 million bases (1Mb) in the tumor genome, and the unit is Muts/Mb. Non-synonymous mutations in TMB include point mutations (SNV), Indel mutations and Splicing mutations, but not copy number variations (CNV), Fusion mutations and germline mutations. According to an official report by Foundation medicine in 2017, TMB is divided into three categories: low TMB (1-5 muTs/Mb), medium TMB (6-19 muTs/Mb), and high TMB (≥20) (17). In this study, considering the low TMB rate in HCC, we divided TMB into low TMB group (≤5Muts/Mb) and high TMB group (> 5Muts/Mb).

*TP53*, *TERT* and other gene mutations were calculated as gene mutation rate (%)/copy number at the DNA level.

### 2.6 Treatment procedure

Immune checkpoint inhibitors (ICIs) were administered intravenously every 3 weeks (**Supplementary Table 2**) and targeted drugs were taken orally daily for 1 year or until disease progression and intolerable adverse reactions occurred. Blood samples were collected before each cycle of ICIs injection. Tumor size alterations were evaluated using enhanced computed tomography or magnetic resonance imaging every six weeks, employing mRECIST criteria. Adverse reactions were evaluated using the Common Terminology Criteria for Adverse Events, version 5.0. If a grade 3/4 treatment-related adverse event occurred, treatment was suspended and then restarted at the discretion of two or more attending physicians.

According to the criteria of mRECIST, complete response (CR) is characterized by the complete disappearance of all target lesions, the absence of any new lesions, and the presence of normal tumor markers for a minimum duration of 4 weeks. Partial response (PR) is defined as a reduction of at least 30% in the combined maximum diameters of all target lesions, sustained for a minimum of 4 weeks. Stable disease (SD) is described as a reduction in the combined maximum diameters of the target lesions that does not meet the criteria for PR, or an increase that does not meet the criteria for progression disease (PD). PD is defined as an increase of at least 20% in the combined maximum diameters of all target lesions, or the appearance of new lesions.

OS was defined as the duration from the initiation of treatment to the death of an individual due to any cause or the most recent follow-up date. PFS was defined as the duration from treatment to recurrence or metastasis of the disease. The ORR, encompassing CR and PR, was established as the percentage of individuals who achieved either CR or PR. Meanwhile, the disease control rate (DCR), comprising CR, PR, and SD, was established as the proportion of individuals who either achieved remission or maintained stability during treatment.

### 2.7 Statistical analysis

SPSS (version 22.0) and R software (version 4.3.1) were utilized for conducting the statistical analysis. Quantitative data that followed a normal distribution were presented as $\bar{X}\pm S$ , and a t-test was employed to compare the two groups. For non-normally distributed quantitative data, they were expressed as [M (P25~P75)], and the Mann-Whitney U test was used for group comparisons. Qualitative data were presented as the number of cases (%) and analyzed using the χ^2^ test for group comparisons.

Kaplan-Meier analysis was used to detect survival differences between the different groups. The Cox proportional hazards regression model was used for multivariate survival analysis, the selected variables were utilized to create nomograms for the prediction of 1-, 2-, and 3-year OS rates. The discriminative power and accuracy of the model were evaluated using calibration curves made by the “rms” and “survival” packages of R software. The time receiver operating characteristic (tROC) curve was employed to determine the optimal cut-off value for prognostic index. The “pheatmap” and “vegan “packages of R software were used for mutation spectrum and cluster analysis, and graphs were made.

In terms of statistical analysis, a significance level of P<0.05 was deemed statistically significant.

## 3 Results

### 3.1 Participant characteristics

The median age of participants was 54.00 years (IQR: 48.0-62.0), with 88.98% of them were male. O-type blood was found in 26.77% of the patients. The majority of patients (81.89%) had an ECOG score of 0. Most participants had Child-Pugh A grade (70.87%) and were classified as BCLC stage B (63.78%). Hepatitis B was identified as the most common cause of HCC (79.53%), and cirrhosis was present in 51.18% of the patients. Median serum AFP was 229.6 ng/mL (IQR: 5.83-3021). Lymphatic metastasis was found in 34 patients (26.77%), and microvascular invasion (MVI) was observed in 72 patients (56.69%). 60.63% of patients had PD-L1 expression (PD-L1>1%). TMB≥5Mb were existed in 33.86% of patients. 58.27% and 54.33% of patients exhibited *TERT* and *TP53* mutations, respectively (**Table 1**).

**Table 1:** Baseline clinical characteristics of the patients.

Variable	All (N=127)
Age, median (IQR), year	54.00 (48-62)
Gender	
Male	113 (88.98%)
Female	14 (11.02%)
ECOG performance score	
0	104 (81.89%)
1	23 (18.11%)
Blood type	
non O-type blood	93 (73.23%)
O-type blood	34 (26.77%)
HBV	
Absent	26 (20.47%)
Present	101 (79.53%)
Cirrhosis	
Absent	62 (48.82%)
Present	65 (51.18%)
Child-Pugh class	
5	40 (31.50%)
6	50 (39.37%)
7	37 (29.13%)
AFP (IQR), ng/mL	229.6 (5.83-3021)
BCLC stage	
B	81 (63.78%)
C	46 (36.22%)
Lymphatic metastasis	
Absent	93 (73.23%)
Present	34 (26.77%)
MVI	
Absent	55 (43.31%)
Present	72 (56.69%)
PD-L1	
Absent	50 (39.37%)
Present	77 (60.63%)
TMB	
≥5 Mb	43 (33.86%)
<5 Mb	84 (66.14%)
*TERT*	
Absent	53 (41.73%)
Present	74 (58.27%)
*TP53*	
Absent	61 (48.03%)
Present	66 (54.33%)

### 3.2 Survival analysis

The median PFS and OS after starting targeted and immunotherapy were 9 months and 21 months, respectively. The ORR was 21.26%. We observed 3 patients (2.36%) achieved CR, 24 patients (18.90%) achieved PR, 48 patients (37.80%) maintained SD and 52 patients (40.94%) were PD.

**Figure 2:**
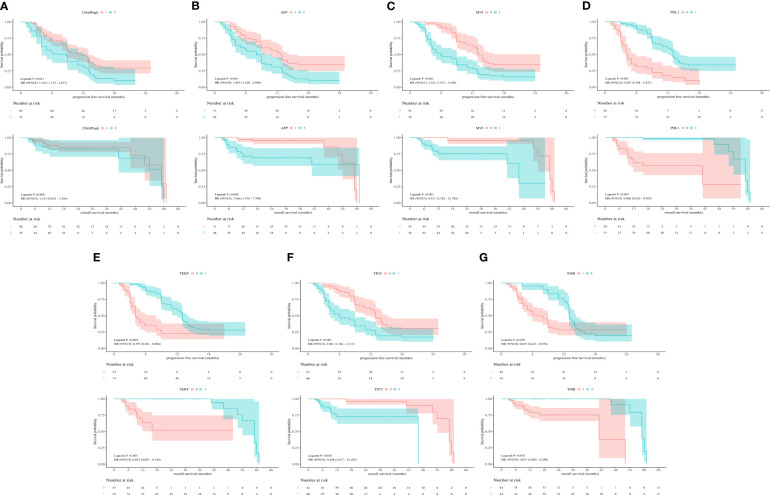
**(A)** Kaplan-Meier survival curves showing PFS and OS according to Child-Pugh class. **(B)** Kaplan-Meier survival curves showing PFS and OS according to AFP. **(C)** Kaplan-Meier survival curves showing PFS and OS according to MVI. **(D)** Kaplan-Meier survival curves showing PFS and OS according to PD-L1. **(E)** Kaplan-Meier survival curves showing PFS and OS according to *TERT* expression. **(F)** Kaplan-Meier survival curves showing PFS and OS according to *TP53* expression. **(G)** Kaplan-Meier survival curves showing PFS and OS according to TMB.

According to the Kaplan-Meier survival curve, Child-Pugh class A versus class B (11 vs 9 mo, P=0.011), AFP< 400 ng/mL versus ≥400 ng/mL(13 vs. 8 mo, p<0.001), non-MVI versus MVI (13 vs. 6 mo, p< 0.001), PD-L1≥1% versus<1% (13 vs. 4 mo, p< 0.001), *TERT* MU versus *TERT* WT(13 vs. 4 mo, p< 0.001), *TP53* WT versus *TP53* MU (13 vs. 6 mo, p< 0.001) and TMB≥5Mb versus< 5Mb (13 vs.7 mo, p=0.039) remarkably prolonged the mPFS in the enrolled individuals. Furthermore, AFP< 400ng/mL versus ≥400 ng/mL (61 vs. 59 mo, p=0.004), non-MVI versus MVI (59 vs. 46 mo, p< 0.001), PD-L1≥1% versus<1% (59 vs. 41 mo, p< 0.001), *TERT* MU versus *TERT* WT (59 vs. NA mo, p< 0.001), *TP53* WT versus *TP53* MU (59 vs. 46 mo, p< 0.001) and TMB≥5Mb versus< 5Mb (59 vs. 41 mo, p =0.001) prolonged the mOS in the whole participants (**Figure 2**).

### 3.3 Construction of a prognostic prediction nomogram

The univariate analysis showed that AFP< 400 ng/mL (HR 1.99, 95% CI 1.32 - 2.99, p< 0.001), non-MVI (HR 0.43, 95% CI 0.28 - 0.66, p< 0.001), PD-L1≥1% (HR3.49, 95%CI 2.29 - 5.31, p< 0.001), TMB≥5Mb (HR 1.57, 95% CI 1.02 - 2.41, p=0.039), *TERT* MU(HR 2.52, 95% CI 1.66 - 3.84, p<0.001) and *TP53* WT (HR 0.49, 95% CI 0.32 - 0.73, p<0.001) were linked to longer PFS. These related single factors were selected for multivariate analysis. The acquired data implied that PD-L1≥1% (HR 1.95, 95% CI 1.14 - 3.33, p = 0.015) and *TERT* MU (HR 2.47, 95% CI 1.49 - 4.08, p<0.001) remained independent prognostic factors for PFS (**Table 2**).

**Table 2:** Univariate and multivariate cox regression analyses for PFS.

Variable	(n=127)	Univariate analysis		Multivariate analysis	
		HR (95%CI)	*P*	HR (95%CI)	*P*
Age (≤55y/>55y)	74/53	1.06 (0.71 - 1.60)	0.765		
Sex (M/F)	113/14	1.45 (0.79 - 2.67)	0.228		
ECOG (0/1)	104/23	1.03 (0.60 - 1.77)	0.912		
Blood type (non-O/O)	93/34	1.37 (0.88 - 2.15)	0.168		
HBV (Absent/Present)	101/26	0.94 (0.56 - 1.57)	0.817		
Cirrhosis (Absent/Present)	65/62	0.96 (0.64 - 1.44)	0.845		
Child-Pugh class (A/B)	90/37	1.74 (1.13 - 2.68)	0.011	1.59 (0.99 - 2.57)	0.057
AFP (<400ng/ml/≥400ng/ml)	71/56	1.99 (1.32 - 2.99)	<0.001	1.32 (0.78 - 2.23)	0.294
BCLC stage (C/B)	81/46	1.10 (0.72 - 1.67)	0.653		
Lymphatic metastasis (Absent/Present)	34/93	0.71 (0.45 - 1.12)	0.143		
MVI (Absent/Present)	72/55	0.43 (0.28 - 0.66)	<0.001	0.70 (0.25 - 1.97)	0.499
PD-L1 (Absent/Present)	50/77	3.49 (2.29 - 5.31)	<0.001	1.95 (1.14 - 3.33)	0.015
TMB (<5/≥5)	84/43	1.57 (1.02 - 2.41)	0.039	1.19 (0.75 - 1.92)	0.459
*TERT* (Absent/Present)	53/74	2.52 (1.66 - 3.84)	<0.001	2.47 (1.49 - 4.08)	<0.001
*TP53* (Absent/Present)	61/66	0.49 (0.32 - 0.73)	<0.001	0.94 (0.39 - 2.27)	0.884

The univariate analysis showed that AFP< 400 ng/mL (HR 3.37, 95% CI 1.47 - 7.71, p = 0.004), non-MVI (HR 0.14, 95% CI 0.05 - 0.46, p<0.001), PD-L1≥1% (HR 15.11, 95% CI 4.94 - 46.20, p< 0.001), TMB≥5Mb (HR 32.49, 95% CI 3.88 - 271.96, p=0.001), *TERT* MU (HR 30.45, 95% CI 6.67 - 138.99, p< 0.001) and *TP53* WT (HR 0.11, 95% CI 0.03 - 0.38, p< 0.001) were associated with longer OS. In multivariate analysis, PD-L1≥1% (HR 25.07, 95%CI 1.56 - 403.29, p=0.023), TMB≥5Mb (HR 86.67, 95% CI 4.00 - 1876.48, p=0.004), *TERT* MU (HR 84.09, 95% CI 5.23 - 1352.70, p=0.002) and *TP53* WT (HR 0.01, 95%CI 0.00 - 0.47, p=0.022) remained independent prognostic factors for OS (**Table 3**).

**Table 3:** Univariate and multivariate cox regression analyses for OS.

Variable	(n=127)	Univariate analysis		Multivariate analysis	
		HR (95%CI)	*P*	HR (95%CI)	*P*
Age (≤55y/>55y)	74/53	1.16 (0.51 - 2.62)	0.729		
Sex (M/F)	113/14	1.65 (0.48 - 5.68)	0.426		
ECOG (0/1)	104/23	2.20 (0.83 - 5.82)	0.112		
Blood type (non-O/O)	93/34	1.91 (0.79 - 4.61)	0.152		
HBV (Absent/Present)	101/26	0.79 (0.27 - 2.33)	0.668		
Cirrhosis (Absent/Present)	65/62	1.01 (0.45 - 2.24)	0.983		
Child-Pugh class (A/B)	90/37	1.41 (0.62 - 3.19)	0.406		
AFP (<400ng/ml/≥400ng/ml)	71/56	3.37 (1.47 - 7.71)	0.004	1.47 (0.56 - 3.86)	0.432
BCLC stage (C/B)	81/46	0.55 (0.23 - 1.34)	0.190		
Lymphatic metastasis (Absent/Present)	34/93	0.67 (0.27 - 1.63)	0.372		
MVI (Absent/Present)	72/55	0.14 (0.05 - 0.46)	<0.001	11.92 (0.39 - 369.05)	0.157
PD-L1 (Absent/Present)	50/77	15.11 (4.94 - 46.20)	<0.001	25.07 (1.56 - 403.29)	0.023
TMB (<5/≥5)	84/43	32.49 (3.88 - 271.96)	0.001	86.67 (4.00 - 1876.48)	0.004
*TERT* (WT/MU)	53/74	30.45 (6.67 - 138.99)	<0.001	84.09 (5.23 - 1352.70)	0.002
*TP53* (WT/MU)	61/66	0.11 (0.03 - 0.38)	<0.001	0.01 (0.00 - 0.47)	0.022

**Figure 3:**
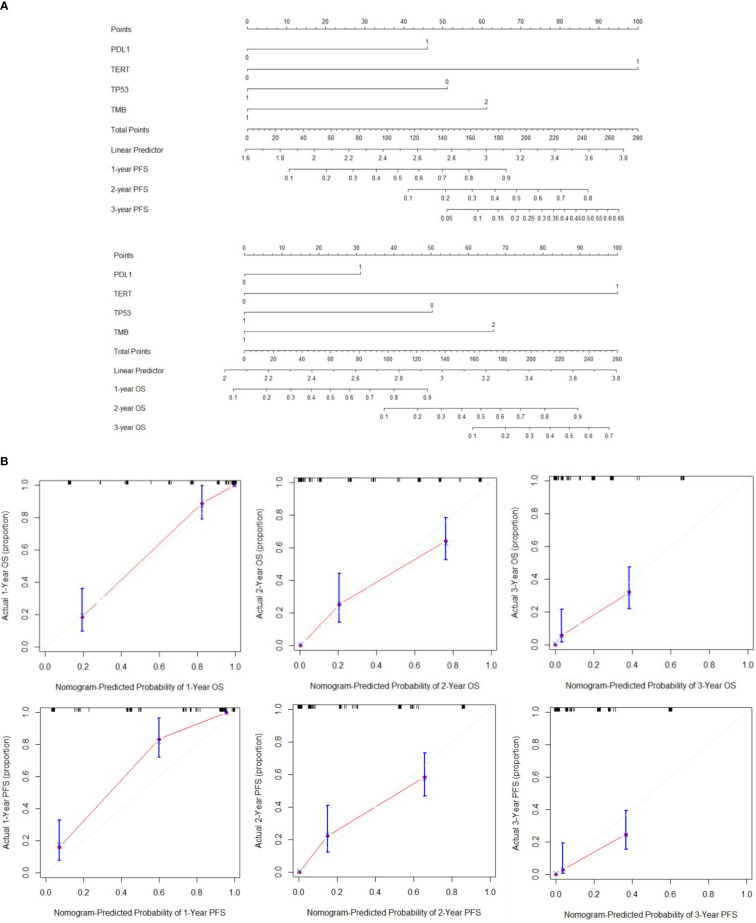
**(A)** Establishment of PFS and OS nomograms. **(B)** The calibration curves for predicting OS and PFS at (A) 1-year and (B) 2-year and (C) 3-year.

We constructed a nomogram based on multivariate Cox regression analysis to predict the 1 -, 2 -, and 3-year survival probabilities of aHCC patients (**Figure 3A**). As shown in the calibration plot, the nomogram robustly predicted PFS and OS in aHCC patients (**Figure 3B**).

### 3.4 Construction of a prognostic index model

**Figure 4:**
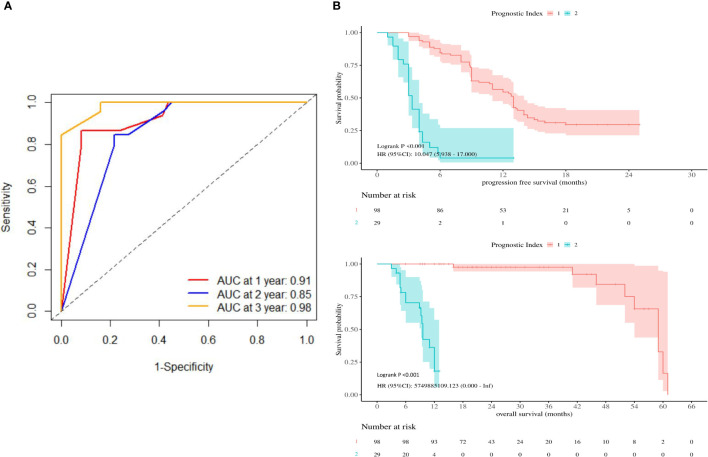
**(A)** Receiver operating characteristic curves were produced at 1-, 2-, and 3-year. **(B)** Kaplan-Meier survival curves showing PFS and OS according to prognostic index.

Based on the expression of relevant genes and Cox proportional hazards model, the survival prognostic index for each sample were calculated as follows: prognostic index = (2.001×PD-L1 expression) + (4.3×*TERT* expression) - (3.149×*TP53* expression) + (4.338 ×TMB). The AUC values at 1-, 2-, and 3-year of follow-up were 0.91, 0.85, and 0.98, respectively (**Figure 4A**), indicating good sensitivity and specificity of this prognostic index. The best cutoff prognostic index is 1.2, dividing the patients into high-risk group and low-risk group. According to the Kaplan-Meier survival curve, high-risk group remarkably had the shorter mPFS(13 vs. 3 mo, P< 0.001) and mOS(59 vs. 9.6 mo, p< 0.001) than low-risk group (**Figure 4B**).

Compared with low-risk group, high-risk group were younger (54.5 vs. 53 y, p=0.033), had higher AFP (33.03 vs. 29959, p< 0.001), higher MVI rate (44.90% vs. 96.55%, p< 0.001), higher PD-L1 negative rate (23.47% vs. 93.10%, p< 0.001), higher TMB< 5 Mb rate (58.16% vs. 96.55%, p< 0.001), higher *TERT* WT rate (26.53% vs. 93.10%, p< 0.001), higher *TP53* MU rate (38.78% vs. 96.55%, p< 0.001) (**Supplementary Table 1**), the result is consistent with our established prognostic prediction model.

**Figure 5:**
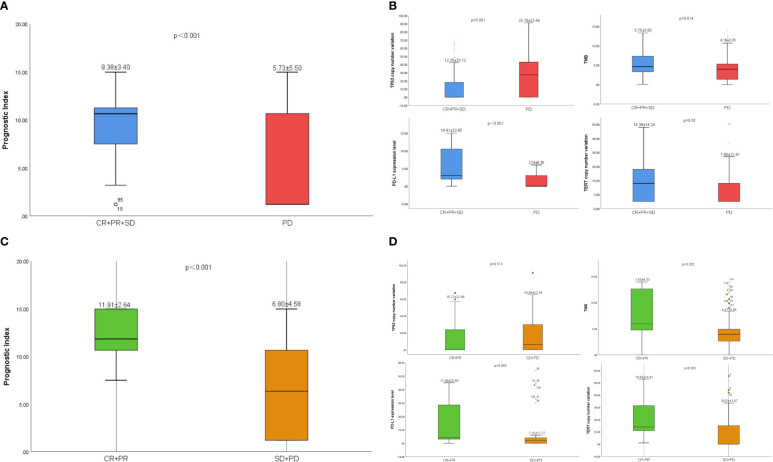
**(A)** The difference of prognostic index between CR/PR/SD group and PD group. **(B)** The difference of *TP53*、TMB、PD-L1 and *TERT* between CR/PR/SD group and PD group. **(C)** The difference of prognostic index between CR/PR group and SD/PD group. **(D)** The difference of *TP53*、TMB、PD-L1 and *TERT* between CR/PR group and SD/PD group.

The prognostic index level in CR/PR/SD group was significantly higher than that in PD group (9.38 vs. 5.73, p< 0.001) (**Figure 5A**), CR/PR group was significantly higher than that in SD/PD group (11.91 vs. 6.80, p< 0.001) (**Figure 5C**). The ORR of the low-risk group was 25.51% and the DCR was 72.45%. The ORR and DCR of high-risk group were 6.90% and 13.79%, respectively.

### 3.5 Treatments and prognostic index

**Figure 6:**
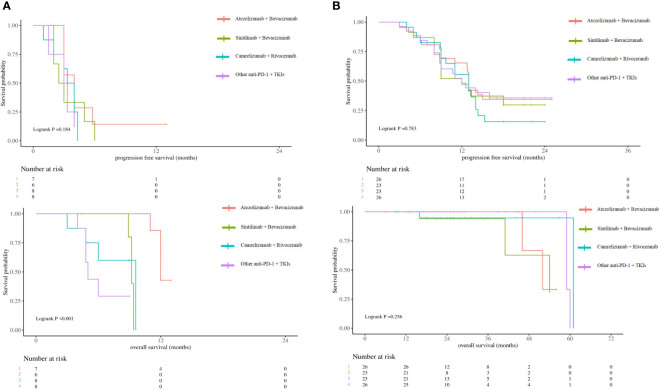
**(A)** Kaplan-Meier survival curves showing PFS and OS according to different combinations of treatments in high-risk group. **(B)** Kaplan-Meier survival curves showing PFS and OS according to different combinations of treatments in low-risk group.

According to the 2023 CSCO guidelines, Atezolizumab + Bevacizumab (3), Sintilimab + Bevacizumab (18), Camrelizumab + Rivoceranib (19) have been approved for the first-line treatment of unresectable aHCC. Survival analyses were stratified according to different combinations of treatments. The results showed that the difference ICIs and targeted drugs did not affect the prognosis of patients in the low-risk group (p>0.05), while Atezolizumab + Bevacizumab could obtain longer OS in the high-risk group (12mo vs 9.2mo vs 9.6mo vs 5mo, p<0.001) (**Figure 6**).

### 3.6 Landscape of genomic alterations

**Figure 7:**
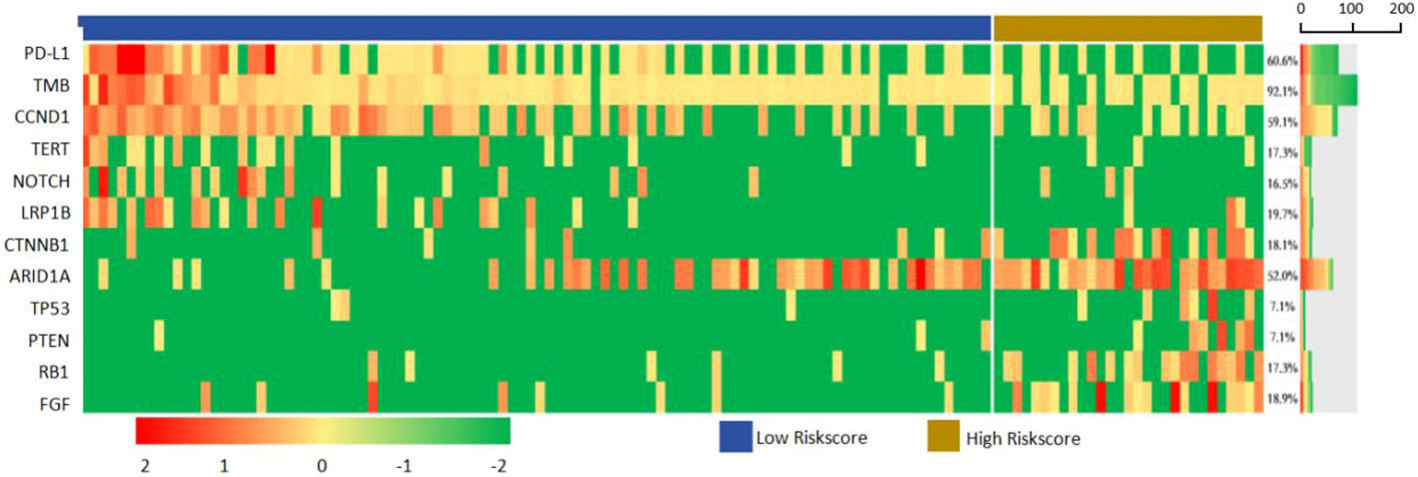
The alteration spectrum of 127 aHCC patients, including *TERT*, *TP53* and CTNNB1 alterations.

**Figure 8:**
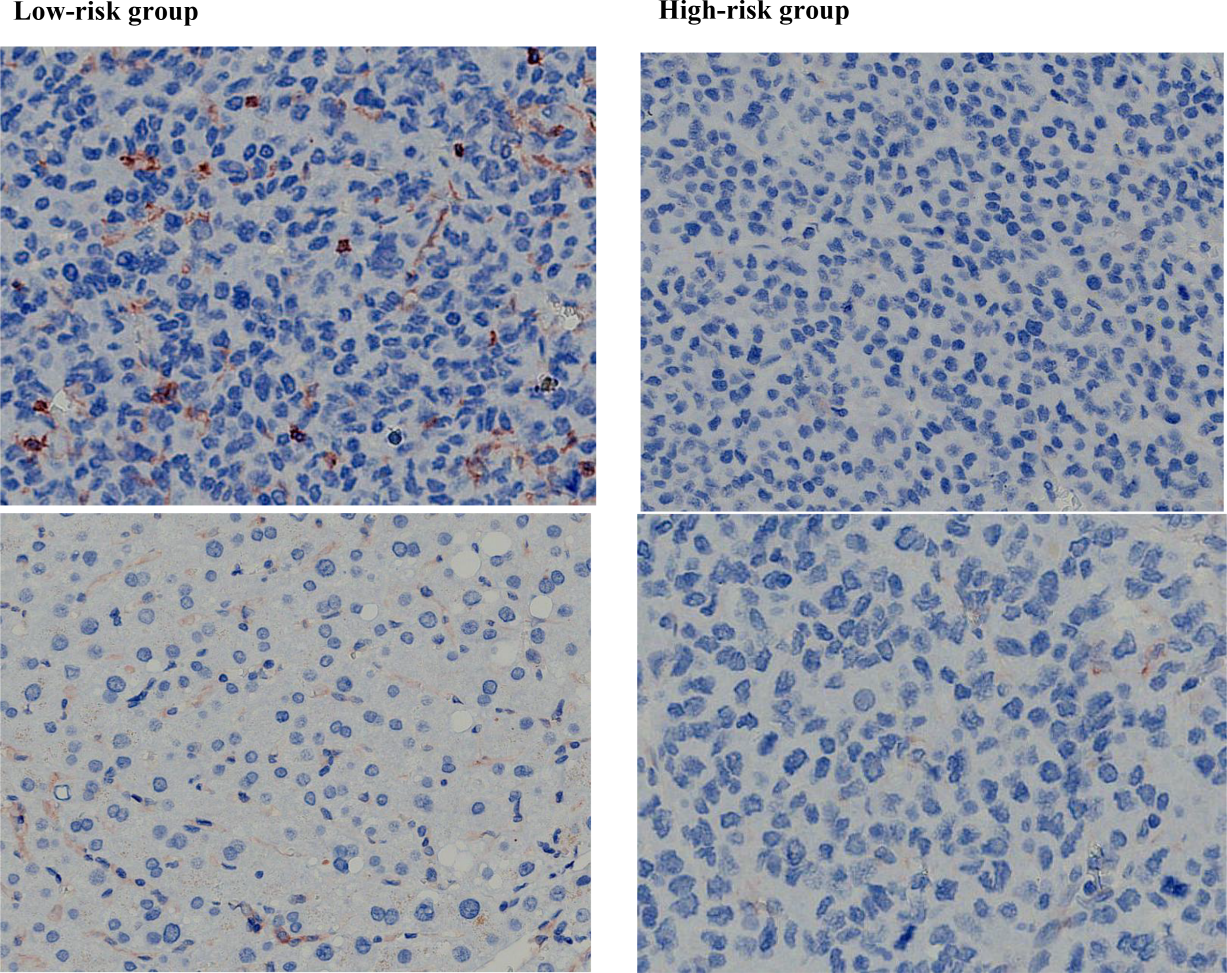
The expression level of PD-L1 in high-risk group was lower than that in low-risk group.

In all samples, the most common mutations including *TERT* (58.27%), *TP53* (54.33%), *ARID1A* (19.69%), *FGF* (18.90%), *CTNNB1* (18.11%), *NOTCH* (17.32%), *CCND* (17.32%), *LRP1B* (16.54%), *PTEN* (7.09%) and *RB1* (7.09%). The above 12 genes were mapped into the gene mutation spectrum of HCC (**Figure 7**). *TERT*, *NOTCH*, *LRP1B* and *ARID1A* were upregulated genes, while *TP53*, *CTNNB1*, *PTEN*, *RB1*, *CCND* and *FGF* were downregulated genes. In addition, the expression of PD-L1 was present by immunohistochemistry (**Figure 8**). The median TMB value of HCC patients was 4.49Mb (IQR: 3.24-6) in our study. The TMB was lower in the high-risk group than that in the low-risk group (5.7 vs. 2.9, p< 0.001).

Clinicopathological features, including age, Child-Pugh class, MVI and AFP were associated with genomic alterations (**Supplementary Table 1**). Advanced age was associated with high TMB (40.5% vs.59.5%, p=0.004) and *TERT* mutation (50.0% vs.50.0%, p=0.026), patients with type O blood more likely had low TMB (67.1% vs.32.9%, p=0.025). MVI was more common in patients with *TP53* mutation (0.0% vs. 100.0%, p< 0.001), PD-L1 negative (10.0% vs. 90.0%, p< 0.001) and *TERT* WT(26.4% vs. 73.6%, p=0.001). Good liver function related to positive PD-L1 expression (80.5% vs. 19.5%, p=0.003). High AFP level was associated with *TP53* mutation (30.3% vs. 69.7%, p< 0.001), negative PD-L1 expression (34.0% vs. 66.0%, p< 0.001), and *TERT* WT (43.4% vs. 56.6%, p=0.016).

The level of PD-L1(14.41 vs. 2.04, p< 0.001)、TMB (5.75 vs. 4.16, p = 0.014) and *TERT* (14.38 vs. 7.90, p=0.01) in CR/PR/SD group was significantly higher than that in PD group. However, the level of *TP53* in CR/PR/SD group was significantly lower than that in PD group (12.26 vs. 25.78, p=0.001) (**Figure 5B**). The same was true in CR/PR group and SD/PD group (**Figure 5D**).

## 4 Discussion

In this study, we showed the NGS practicability in targeted and immunotherapy in advanced HCC clinical practice. Consistent with previous literature reports, high-frequency mutations such as *TP53* and *CTNNB1* are closely associated with high TMB (20), and patients carrying *TP53* neoantigens have longer OS than other patients (21). Mutations in *TERT*, *CTNNB1*, *BRD4* or *MLL*, as well as co-mutations in *TP53* and *TERT* or *BRD4*, were associated with significantly worse survival (22). In addition, some studies have shown that patients with high TMB exhibit worse OS than those with low TMB (23). However, the above studies are from first-line or multi-line single-agent immunotherapy, and the stratification effect of mutation and TMB on response and prognosis in first-line targeted and immunotherapy has not been reported. We found that *TERT* mutations were associated with poor prognosis, while PD-L1, TMB, and *TP53* were associated with response to immune checkpoint blockade in patients with advanced HCC treated with targeted and immunotherapy.

In addition, we identified 12 genes with stratification ability for first-line targeted and immunotherapy. These genes were mainly involved in liver metabolic regulation and tumor immune regulation. *NOTCH*, *LRP1B*, *ARID1A* are upregulated genes, *CTNNB1*, *PTEN*, *RB1*, *CCND* and *FGF* are downregulated genes. *CTNNB1* mutant tumors have low immune infiltration, oncogenic WNT signaling leading to t lymphocyte rejection (24), and are insensitive to combination therapy with anti-PD-L1 and anti-CTLA-4 monoclonal antibodies *in vivo* (25). Some studies indicated that all carried activated *CTNNB1* mutations in tumor patients have disease progression, and non-WNT pathways changes of tumor patients were significantly more likely to respond or gain clinical benefit from immunotherapy (14). Notch pathway activation is associated with poor response and shorter PFS (26). Li et al. reported that *ARID1A* mutation in gastrointestinal cancer was associated with high TMB and PD-L1 positive expression, and speculated that *ARID1A* mutation may be used as a biomarker to identify the efficacy of immunotherapy (27). *FGF3/4/19* amplification has also been reported as a predictive marker of cancer highly progressive disease (HPD) in a variety of treatment lines for HCC and other cancers (28).

Thus, these gene mutations reflect abnormal changes in metabolism and immune regulation in HCC and are significantly associated with response. Thus, these 12 genes constitute a panel that can be used as predictors of response to first-line targeted and immunotherapy. Due to the low mutation rate of most genes and the limited number of patients, these 12 genes were not all validated in this study, and subsequent studies should model and validate them.

The significance of this study is that most aHCC patients can be correctly stratified according to NGS results before treatment initiation, and patients in the high-risk group will achieve longer survival with Atezolizumab + Bevacizumab. To the best of our knowledge, this finding has not been previously reported. By analyzing the relationship between clinical characteristics and NGS results, we found that, first, advanced age was associated with high TMB and *TERT* mutations. This is consistent with previous studies showing that TMB increases significantly with age, showing a 2.4-fold difference between age 10 and age 90 years (29). Second, in our study, patients with O-type blood more likely to have low TMB. A number of studies showed that HCC prognosis is associated with blood type, patients with non-O type blood, particularly AB-type blood, had a worse OS compared with those having O-type blood (30). Third, MVI concentration in patients with *TP53* mutations. The mutant incidence of genes like *KEAP1*, *TP53*, *HIST1H3D*, *NFKBIA*, *PIK3CB*, and *WRN* was higher in both MVI and early-recurrence patients than their counterparts (31). Fourth, a good liver function associated with PD-L1 positive expression. The immune and metabolic microenvironment is of great significance for the occurrence and development of HCC, and PD1/PD-L1, as a link in the immune response, may be involved in the progression of chronic liver disease (32). Fifth, high AFP level is associated with *TP53* mutation. AFP was also identified as the target gene of the p53 transcription factor (33).

This study has certain limitations. First, the number of aHCC patients receiving first-line targeted and immunotherapy is limited, so only 151 patients were included in this study. This limits the ability to perform stratified analyses by mutational, amplified sequencing, and there may be bias in a few cases. Secondly, the number of tumor tissues obtained by puncture is limited, and the clonal complexity of tumors cannot be fully evaluated, which affects the authenticity of sequencing results. Third, because of single-agent immunotherapy is inferior to combination therapy, our overall study of targeted and immunotherapy may reduce the stratification effect of individual mutations and the improved overall response may mask the effect of biomarkers. Lastly, due to our targeted assay design, which could have missed viral HBV DNA sequences.

## 5 Conclusions

The high heterogeneity of HCC is a major obstacle in proposing a standard targeted and immunotherapy strategy for aHCC. Therefore, the future of HCC treatment will be individualized management. It is critical to identify driver alterations in the genome, pathways, and tumor microenvironment in each HCC patient. NGS is an ideal tool to aid in such personalized decision making. With the continuous breakthrough of biotechnology, NGS technology is likely to become more convenient and accurate, providing a solid foundation for the selection of systemic treatment for liver cancer and achieving more individualized treatment. However, further preclinical studies and clinical trials are still needed to explore the therapeutic effects of these targeted pathways. It is difficult to obtain reproducible and reliable results due to the complex malignant subtypes and large intra-tumor and inter-tumor heterogeneity of liver cancer. Therefore, it is necessary to carry out more larger sample size of global multicenter, in order to better understand the treatment and prognosis of NGS potential. In the future, genetic testing may become a routine procedure in the systemic treatment of HCC.

## Data availability statement

The original contributions presented in the study are publicly available. This data can be found here: NCBI, accession number PRJNA1096367.

## Ethics statement

The studies involving humans were approved by Tongji Hospital’s Ethics Committee (TJ-IRB20230866). The studies were conducted in accordance with the local legislation and institutional requirements. The participants provided their written informed consent to participate in this study.

## Author contributions

JD: Writing – original draft, Writing – review & editing. EZ: Conceptualization, Investigation, Writing – original draft. ZH: Conceptualization, Data curation, Investigation, Writing – review & editing.

## Supplementary material

Supplementary Table 1 Clinical characteristics of high-risk group and low-risk group.

Supplementary Table 2 The product name, dosage of ICIs.

## References

[1] Siegel RL Miller KD Fuchs HE Jemal A. Cancer statistics, 2021. CA Cancer J Clin. (2021) 71:7–33. doi: 10.3322/caac.21654 33433946

[2] Yang JD Hainaut P Gores GJ Amadou A Plymoth A Roberts LR. A global view of hepatocellular carcinoma: trends, risk, prevention and management. Nat Rev Gastroenterol Hepatol. (2019) 16:589–604. doi: 10.1038/s41575-019-0186-y 31439937 PMC6813818

[3] Cheng AL Qin S Ikeda M Galle PR Ducreux M Kim TY. Updated efficacy and safety data from IMbrave150: Atezolizumab plus bevacizumab vs. sorafenib for unresectable hepatocellular carcinoma. J Hepatol. (2022) 76:862–73. doi: 10.1016/j.jhep.2021.11.030 34902530

[4] Kudo M Finn RS Edeline J Cattan S Ogasawara S Palmer DH. Updated efficacy and safety of KEYNOTE-224: a phase II study of pembrolizumab in patients with advanced hepatocellular carcinoma previously treated with sorafenib. Eur J Cancer. (2022) 167:1–12. doi: 10.1016/j.ejca.2022.02.009 35364421

[5] Yau T Kang YK Kim TY El-Khoueiry AB Santoro A Sangro B. Efficacy and safety of nivolumab plus ipilimumab in patients with advanced hepatocellular carcinoma previously treated with sorafenib: the checkMate 040 randomized clinical trial. JAMA Oncol. (2020) 6:e204564. doi: 10.1001/jamaoncol.2020.4564 33001135 PMC7530824

[6] Schulze K Imbeaud S Letouzé E Alexandrov LB Calderaro J Rebouissou S. Exome sequencing of hepatocellular carcinomas identifies new mutational signatures and potential therapeutic targets. Nat Genet. (2015) 47:505–11. doi: 10.1038/ng.3252 PMC4587544 25822088

[7] Fulgenzi CAM Cheon J D’Alessio A Nishida N Ang C Marron TU. Reproducible safety and efficacy of atezolizumab plus bevacizumab for HCC in clinical practice: Results of the AB-real study. Eur J Cancer. (2022) 175:204–13. doi: 10.1016/j.ejca.2022.08.024 36148739

[8] Liao W Yang H Xu H Wang Y Ge P Ren J. Noninvasive detection of tumor-associated mutations from circulating cell-free DNA in hepatocellular carcinoma patients by targeted deep sequencing. Oncotarget. (2016) 7:40481–90. doi: 10.18632/oncotarget.9629 PMC5130021 27248174

[9] Lazarus DR Ost DE. How and when to use genetic markers for nonsmall cell lung cancer. Curr Opin Pulm Med. (2013) 19:331–9. doi: 10.1097/MCP.0b013e328362075c PMC3926417 23715289

[10] Garufi G Palazzo A Paris I Orlandi A Cassano A Tortora G. Neoadjuvant therapy for triple-negative breast cancer: potential predictive biomarkers of activity and efficacy of platinum chemotherapy, PARP- and immune-checkpoint-inhibitors. Expert Opin Pharmacother. (2020) 21:687–99. doi: 10.1080/14656566.2020.1724957 32052646

[11] Bang YJ Golan T Dahan L Fu S Moreno V Park K. Ramucirumab and durvalumab for previously treated, advanced non-small-cell lung cancer, gastric/gastro-oesophageal junction adenocarcinoma, or hepatocellular carcinoma: An open-label, phase Ia/b study (JVDJ). Eur J Cancer. (2020) :137:272–284. doi: 10.1016/j.ejca.2020.06.007 32827847

[12] Yau T Park JW Finn RS Cheng AL Mathurin P Edeline J. Nivolumab versus sorafenib in advanced hepatocellular carcinoma (CheckMate 459): a randomised, multicentre, open-label, phase 3 trial. Lancet Oncol. (2022) 23:77–90. doi: 10.1016/S1470-2045(21)00604-5 34914889

[13] Riaz N Havel JJ Makarov V Desrichard A Urba WJ Sims JS. Tumor and microenvironment evolution during immunotherapy with nivolumab. Cell. (2017) 171:934–49. doi: 10.1016/j.cell.2017.09.028 PMC5685550 29033130

[14] Harding JJ Nandakumar S Armenia J Khalil DN Albano M Ly M. Prospective genotyping of hepatocellular carcinoma: clinical implications of next-generation sequencing for matching patients to targeted and immune therapies. Clin Cancer Res. (2019) 25:2116–26. doi: 10.1158/1078-0432.CCR-18-2293 PMC6689131 30373752

[15] Guo G Yu M Xiao W Celis E Cui Y. Local activation of p53 in the tumor microenvironment overcomes immune suppression and enhances antitumor immunity. Cancer Res. (2017) 77:2292–305. doi: 10.1158/0008-5472.CAN-16-2832 PMC5465961 28280037

[16] Matsumae T Kodama T Myojin Y Maesaka K Sakamori R Takuwa A. Circulating cell-free DNA profiling predicts the therapeutic outcome in advanced hepatocellular carcinoma patients treated with combination immunotherapy. Cancers (Basel). (2022) 14:3367. doi: 10.3390/cancers14143367 35884434 PMC9320668

[17] Goodman AM Kato S Bazhenova L Patel SP Frampton GM Miller V. Tumor mutational burden as an independent predictor of response to immunotherapy in diverse cancers. Mol Cancer Ther. (2017) 16:2598–608. doi: 10.1158/1535-7163.MCT-17-0386 PMC5670009 28835386

[18] Ren Z Xu J Bai Y Xu A Cang S Du C. Sintilimab plus a bevacizumab biosimilar (IBI305) versus sorafenib in unresectable hepatocellular carcinoma (ORIENT-32): a randomised, open-label, phase 2-3 study. Lancet Oncol. (2021) 22:977–90. doi: 10.1016/S1470-2045(21)00252-7 34143971

[19] Qin SK Chan SL Gu S Bai Y Ren Z Lin X. Camrelizumab (C) plus rivoceranib (R) vs. sorafenib (S) as first-line therapy for unresectable hepatocellular carcinoma (uHCC): A randomized, phase III trial. Lancet. (2023) 402(10408):1133-46. doi: 10.1016/j.annonc.2022.08.032 37499670

[20] Li L Rao X Wen Z Ding X Wang X Xu W. Implications of driver genes associated with a high tumor mutation burden identified using next-generation sequencing on immunotherapy in hepatocellular carcinoma. Oncol Lett. (2020) 19:2739–48. doi: 10.3892/ol.2020.11372 PMC7068659 32218826

[21] Yang H Sun L Guan A Yin H Liu M Mao X. Unique TP53 neoantigen and the immune microenvironment in long-term survivors of Hepatocellular carcinoma. Cancer Immunol Immunother. (2021) 70:667–77. doi: 10.1007/s00262-020-02711-8 PMC10992148 32876735

[22] Ou Q Yu Y Li A Chen J Yu T Xu X. Association of survival and genomic mutation signature with immunotherapy in patients with hepatocellular carcinoma. Ann Transl Med. (2020) 8:230. doi: 10.21037/atm.2020.01.32 32309377 PMC7154492

[23] Wong CN Fessas P Dominy K Mauri FA Kaneko T Parcq PD. Qualification of tumour mutational burden by targeted next-generation sequencing as a biomarker in hepatocellular carcinoma. Liver Int. (2021) 41:192–203. doi: 10.1111/liv.14706 33098208

[24] Xiao Q Wu J Wang WJ Chen S Zheng Y Yu X. DKK2 imparts tumor immunity evasion through β-catenin-independent suppression of cytotoxic immune-cell activation. Nat Med. (2018) 24:262–70. doi: 10.1038/nm.4496 PMC5840007 29431745

[25] Spranger S Bao R Gajewski TF. Melanoma-intrinsic β-catenin signalling prevents anti-tumour immunity. Nature. (2015) 523:231–5. doi: 10.1038/nature14404 25970248

[26] Zhu AX Guan Y Abbas AR Koeppen H Lu S Hsu C-H. Abstract CT044: genomic correlates of clinical benefits from atezolizumab combined with bevacizumab vs. atezolizumab alone in patients with advanced hepatocellular carcinoma (HCC). Cancer Res. (2020) 80:CT044–4. doi: 10.1158/1538-7445.Am2020-ct044

[27] Li L Li M Jiang Z Wang X. ARID1A mutations are associated with increased immune activity in gastrointestinal cancer. Cells. (2019) 8:678. doi: 10.3390/cells8070678 31277418 PMC6678467

[28] Li F Li Z Han Q Cheng Y Ji W Yang Y. Enhanced autocrine FGF19/FGFR4 signaling drives the progression of lung squamous cell carcinoma, which responds to mTOR inhibitor AZD2104. Oncogene. (2020) 39:3507–21. doi: 10.1038/s41388-020-1227-2 PMC7176586 32111983

[29] Chalmers ZR Connelly CF Fabrizio D Gay L Ali SM Ennis R. Analysis of 100,000 human cancer genomes reveals the landscape of tumor mutational burden. Genome Med. (2017) 9:34. doi: 10.1186/s13073-017-0424-2 28420421 PMC5395719

[30] Li Q Wu T Ma XA Jing L Han LL Guo H. Prognostic role of ABO blood group in patients with unresectable hepatocellular carcinoma after transarterial chemoembolization. Ther Clin Risk Manage. (2018) 14:991–8. doi: 10.2147/TCRM.S160089 PMC5985783 29881281

[31] Xin Z Li J Zhang H Zhou Y Song J Chen P. Cancer genomic alterations can be potential biomarkers predicting microvascular invasion and early recurrence of hepatocellular carcinoma. Front Oncol. (2022) 12:783109. doi: 10.3389/fonc.2022.783109 35155229 PMC8828586

[32] Hassan M Attia MS Ali-Eldin Z El Attar G Elzallat M Saad HHK. Programmed death-ligand 1 (PD-L1) polymorphisms as predictive biomarkers for the development of liver cirrhosis and hepatocellular carcinoma in HCV Egyptian patients. Tumour Virus Res. (2022) 14:200249. doi: 10.1016/j.tvr.2022.200249 36265835 PMC9594630

[33] Li H Liu Y Jiang W Xue J Cheng Y Wang J. Icaritin promotes apoptosis and inhibits proliferation by down-regulating AFP gene expression in hepatocellular carcinoma. BMC Cancer. (2021) 21:318. doi: 10.1186/s12885-021-08043-9 33765973 PMC7992931

