# Supplementary material for: The predictive value of next generation sequencing for matching advanced hepatocellular carcinoma patients to targeted and immunotherapy
Source: Front Immunol. 2024 Apr 11;15:1358306. doi: 10.3389/fimmu.2024.1358306 (PMC11043782; doi:10.3389/fimmu.2024.1358306)
Supplement: Supplementary Table 1 — Clinical characteristics of high-risk group and low-risk group. [file Table_1.pdf]

Supplemental Table 1. Clinical characteristics of high-risk group and low-risk group.

Variable	All（N=127）	LRisk(n=98)	HRisk(n=29)	t/χ^2	p
Age, median (IQR), year	54.00（48-62）	54.5(48-63)	53(34.5-61)	2.214	0.033
Gender				1.481	0.224
Male	113（88.98%）	89(90.82%)	24(82.76%)		
Female	14（11.02%）	9(9.18%)	5(17.24%)		
ECOG performance score				0.921	0.337
0	104（81.89%）	82(83.67%)	22(75.86%)		
1	23（18.11%）	16(16.33%)	7(24.14%)		
Blood type				0.013	0.910
non O-type blood	93（73.23%）	72(73.47%)	21(72.41%)		
O-type blood	34（26.77%）	26(26.53%)	8(27.59%)		
HBV				1.030	0.310
Absent	26（20.47%）	22(22.45%)	4(13.79%)		
Present	101（79.53%）	76(77.55%)	25(86.21%)		
Cirrhosis				1.445	0.229
Absent	62（48.82%）	45(45.92%)	17(58.62%)		
Present	65（51.18%）	53(54.08%)	12(41.38%)		
Child-Pugh class				2.730	0.099
5	40（31.50%）	32(32.65%)	8(27.59%)		
6	50（39.37%）	41(41.84%)	9(31.03%)		
7	37（29.13%）	25(25.51%)	12(41.38%)		
AFP (IQR), ng/mL	229.6（5.83-3021）	33.03(3.645-877.175)	29959(1123-60500)	5.126	＜0.001
BCLC stage				2.375	0.123

B	81（63.78%）	59(60.20%)	22(75.86%)		
C	46（36.22%）	39(39.80%)	7(24.14%)		
Lymphatic metastasis				1.140	0.286
Absent	93（73.23%）	74(75.51%)	19(65.52%)		
Present	34（26.77%）	24(24.49%)	10(34.48%)		
MVI				24.318	＜0.001
Absent	55（43.31%）	54(55.10%)	1(3.45%)		
Present	72（56.69%）	44(44.90%)	28(96.55%)		
PD-L1				45.458	＜0.001
Absent	50（39.37%）	23(23.47%)	27(93.10%)		
Present	77（60.63%）	75(76.53%)	2(6.90%)		
TMB				14.899	＜0.001
≥5 Mb	42（33.07%）	41(41.84%)	1(3.45%)		
＜5 Mb	85（66.93%）	57(58.16%)	28(96.55%)		
TERT				40.786	＜0.001
Absent	53（41.73%）	26(26.53%)	27(93.10%)		
Present	74（58.27%）	72(73.47%)	2(6.90%)		
TP53				29.926	＜0.001
Absent	61（48.03%）	60(61.22%)	1(3.45%)		
Present	66（54.33%）	38(38.78%)	28(96.55%)		

Supplemental Table 2. The product name, dosage of ICIs.

ICIs	dosage
Tislelizumab	200mg，Q3w
Camrelizumab	200mg，Q3w
Sintilimab	200mg，Q3w
Toripalimab	240mg，Q3w
Pembrolizumab	200mg，Q3w
Atezolizumab	1200mg，Q3w
Cadonilimab	10mg/kg，Q3w
Durvalumab	1500mg，Q3w
